# An 18-month-old girl with Vici syndrome: A case report study

**DOI:** 10.1016/j.ymgmr.2025.101205

**Published:** 2025-03-13

**Authors:** Parsa Forouhar, Mohammad Amin Eghtedari, Maryam Taraz, Mahsa Abdullahpour, Mohammad Mehrpouyan, Zhale Askarzade

**Affiliations:** a Research Committee, Dezful University of Medical Sciences, Dezful, Iran; b Medical Faculty, Dezful University of Medical Sciences, Dezful, Iran; c Ahvaz Jundishapur University of Medical Sciences, Ahvaz, Iran.

**Keywords:** Vici syndrome, Albinism, Bilateral cataracts, corpus callosum, Seizures, Cardiac rhabdomyoma, Pediatric

## Abstract

We report an 18-month-old female diagnosed with Vici syndrome, a rare congenital disorder characterized by developmental delay, albinism, cataracts, agenesis of the corpus callosum, hypotonia, and immunological anomalies. The patient, the second child of healthy first-cousin parents, presented with hypotonia at birth and subsequently developed bilateral cataracts, feeding difficulties, and multiple systemic manifestations, including kidney, bladder, and gallstones, as well as cardiomegaly and seizures. Genetic testing identified homozygous mutations in the EPG5 gene, confirming the diagnosis of Vici syndrome. The cardiac evaluation revealed a unique finding: serial echocardiograms initially detected multiple masses, which later regressed spontaneously, suggestive of a cardiac rhabdomyoma.

## Highlights

• We present a detailed case of an 18-month-old girl diagnosed with VICI Syndrome, characterized by developmental delay, albinism, bilateral cataracts, agenesis of the corpus callosum, and immunodeficiency.
• Notably, cardiac evaluation revealed transient echogenic masses suggestive of cardiac rhabdomyoma, a unique cardiac manifestation associated with VICI Syndrome.

## 1 Introduction

Vici syndrome is a rare disease in children whose inheritance is transmitted autosomal-recessive [1]. A mutation in EPG5 causes this disease; the ectopic P granule protein 5 genes on chromosome 18, which which ensure autophagosome-lysosome fusion specifity in the autophagosome-lysosome machinery. This disease was first reported in 1988, and it has a series of disorders, including skin hypopigmentation, bilateral cataracts, corpus callosum agenesis, cleft lip and palate, and combined immunodeficiency [2].

The patients have typically been shown with a wide range of clinical features of Vici syndrome. These contain microcephaly, distinctive facial features, cardiomyopathy, and various others. Further features like hearing impairment, underdeveloped lungs, and renal tubular dysfunction have also been reported in specific instances, contributing to a comprehensive comprehension of the condition [2].

Vici syndrome presents a wide range of symptoms characterized by the main features of agenesis of the corpus callosum, cataracts, ocular hypopigmentation, cardiomyopathy, and immunodeficiency. Growth disorders and acquired microcephaly are key features of this syndrome, indicating the presence of a degenerative (neurological) component in development. In more advanced stages, this syndrome can affect multiple organs, impacting the lungs, thyroid, liver, and kidneys, all of which reduce the life expectancy of patients. Vici syndrome is a member of a new group of hereditary neurometabolic diseases known as congenital disorders of autophagy [3].

This study aims to report a case of an 18-month-old girl who was initially suspected of having Vici syndrome at Ganjavian Hospital in Dezful. She has been subjected to further examination for definitive diagnosis and treatment.

## 2 Case presentation

**Fig. 1:**
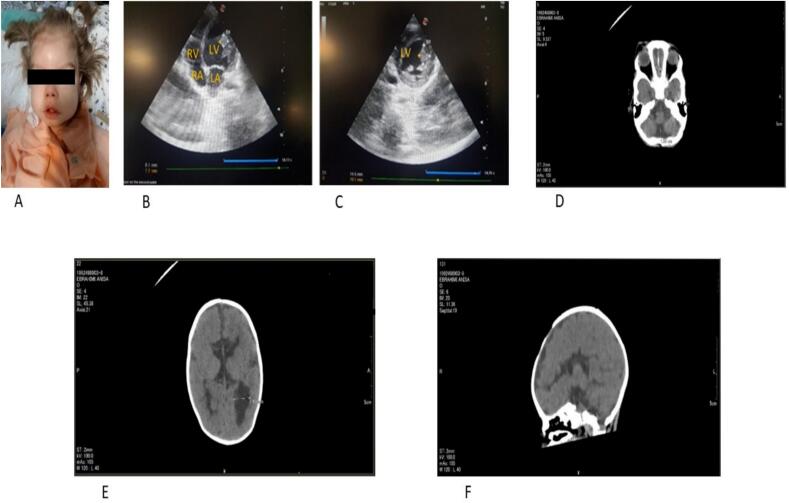
(B and C): Transthoracic echocardiogram in 4 chamber (B) and short axis view (C) RV: right ventricular, LV: left ventricular, LA: left atrium, RA: right atrium. D: Axial view, Focal enlargement of the CSF-filled subarachnoid space is seen in the posteroinferior portion of the posterior cranial fossa, consistent with enlarged cisterna magna maybe due to hypoplasia or agenesis of the vermis. E: axial view. F: sagittal view. E, F: The anterior horns of lateral ventricles are small, with parallel widely separated bodies - the “racing car” sign, with the third ventricle sited highly. There is dilation of the occipital horns of the lateral ventricles, corresponding to colpocephaly. The corpus callosum and septum pellucidum are absent.

We present the case of an 18-month-old girl, the second child in her family, with a healthy male elder sibling. She was born to healthy first-cousin parents, a 27-year-old mother, and a 33-year-old father. The patient was delivered at 38 weeks via natural delivery with an O-negative blood type. She presented with hypotonia and had a birth weight of 3200 g (current weight: 5100 g), a head circumference of 33 cm (current: 39 cm), and a length of 50 cm (current: 69 cm) (Fig. 1 A). No NICU admission was required, and she was discharged shortly after birth. No reported family history of illness or anomalies. The patient was breastfed until two months of age. At that time, her mother noticed she was not tracking objects, leading to a diagnosis of bilateral cataracts. Concurrently, she experienced milk aspiration and nasal regurgitation, necessitating the placement of an NG tube. Her hypotonia became more pronounced.

By six months, the patient exhibited multiple symptoms: albinism, bilateral cataracts, hypotonia, agenesis of the corpus callosum, kidney and bladder stones, gallstones, aspiration, seizures, and cardiomegaly. From six to thirteen months, she received speech and occupational therapy, showing some improvement in hand movements. However, uncontrolled seizures impeded progress, leading to the placement of a PEG tube. Seizures involved the patient's eyes rolling back and her body becoming motionless and tense, initially lasting about one minute but now reduced to thirty seconds, occurring approximately twenty times daily. The patient's eyes react to light, and she has a high-arched palate without a cleft palate. Her fingers and toes appear normal.

Due to the multifactorial nature of this disease, further examinations will be conducted on a section-by-section and organ-by-organ basis, which will be presented to you separately.

### 2.1 Cardiac findings

#### 2.1.1 Transthoracic echocardiography (TTE)

In transthoracic echocardiography (TTE) at five months, the patient exhibited a sizeable left ventricle with an ejection fraction (EF) of 40–45 %, and at eight months, there was a progression to left ventricular systolic and diastolic dysfunction, mild mitral regurgitation (MR) and tricuspid regurgitation (TR), left coronary dilation, and a persistently reduced EF of 40–45 %. At 18 months: Normal visceral situs, mild to moderate enlarged left ventricle and atrium, left ventricular systolic and diastolic dysfunction, EF 40–45 %, mild MR and TR.

A pedunculated mass adjacent to the apex of the left ventricle, measuring 8.1 × 7.7 mm in the 4-chamber view (Fig. 1 B) and 14.5 × 10.1 mm in the short-axis view (Fig. 1 C), was identified alongside two normal papillary muscles. The mass appeared echogenic, homogeneous, non-encapsulated, with a regular border, and showed no signs of calcification, fibrosis, pericardial, or pleural effusion.

Multiple heart masses were first identified in the initial echocardiography performed by another pediatric cardiologist at five months of age. However, no mass was observed in echocardiography performed at eight months. Subsequently, at 18 months, due to the unavailability of cardiac MRI at our center, the initial report of a mass prompted consecutive echocardiograms over two weeks, with no mass detected on the final echocardiogram.

#### 2.1.2 Abdominal and pelvic findings

At five months, the patient presented with a normal liver and gallstones measuring 6.6 mm and 3.5 mm, respectively, without signs of acute inflammation. The kidney parenchyma appeared normal, with a 2.4 mm stone in the left kidney's middle calyx, along with three bladder stones measuring 4.5 mm, 4 mm, and 3 mm in Ultrasounds. By seven months, no kidney enlargement was observed. Hyper-echoic points were noted in the renal collecting systems, with the largest measuring 2 mm, and the bladder appeared normal. At 12 months, the liver was expected, with two gallstones measuring 6 mm and 2.5 mm, again without acute inflammation. The kidney parenchyma remained standard, with a 2.4 mm stone detected in the lower calyces of the left kidney. At 18 months, the liver was normal, but the gallbladder was semi-contracted with a 7 mm stone and evidence of biliary sludge, while the bile ducts appeared normal. There was an increased kidney parenchymal echo, although no stones were observed.

#### 2.1.3 Neurological findings

Axial and Sagittal sections of the patient's CT scan reveal dilation of the occipital horn of the lateral ventricles, predominantly on the left side (Fig. 1 E). This finding indicates agenesis of the corpus callosum, further confirmed by its apparent absence in sagittal sections (Fig. 1 F). A broad cerebral sulcus is consistent with this diagnosis of agenesis of the corpus callosum. Additionally, there is an enlargement of the cisterna magna, measuring up to 15 mm, which suggests hypoplasia or agenesis of the vermis (Fig. 1 D).

Although MRI was recommended for further evaluation, it was deemed impractical due to the patient's unstable condition.

#### 2.1.4 Hearing tests

**Fig. 2:**
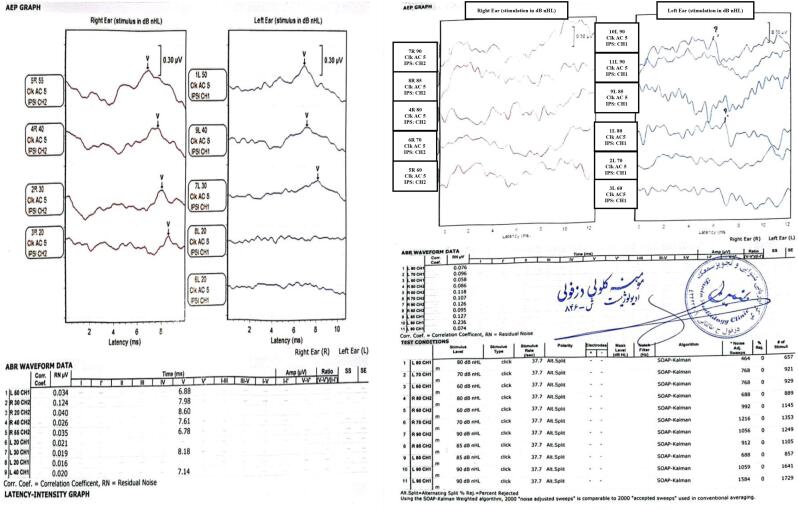
A: ABR: V wave in the left ear up to 20db intensity and in the right ear up to 30db intensity was recorded. B: Auditory brainstem response (ABR) testing revealed that the V wave was examined on both sides with intensities up to 90 dB. No repeatable waves were observed, and the morphology of the waves appeared completely irregular.

No anatomical or pathological abnormalities were detected in the middle or external ear. At one month, auditory brainstem response (ABR) testing indicated normal hearing in the right ear and mild sensorineural hearing loss (SNHL) in the left ear, with a V wave recorded up to 30 dB (Fig. 2 A).

Subsequent ABR testing at 18 months revealed bilateral profound SNHL (Fig. 2 B).

#### 2.1.5 Genetic and metabolic testing

Genetic Tests at 8 months (Table 1):

**Table 1:** Genetic Tests at 8 months.

Analysis of exome data							Immunological Tests Lymphocyte subset quantification at 3 months			The examination of amino acids at the age of 2 and 3 months		
Gene / Transcript	Variant Location	Variant	Chromosome Position (GRCh37)	Zygosity	Related Phenotypes	Inheritance Pattern	CD Markers	Percent (%)	Reference Values	Test	Result	Reference Intervals (Sex and Age adjusted)
EPEG5 ENST00000282041.5 NM_020964	Exon 32	c.5545 > T p.E1849	Chr18: 43,460,162	Hom	VICI syndrome	AR	CD3	49 %	53–84 %	**2 Months**		
PITX3 ENST00000370002.3 NM_005029	Exon 4	c.640G > A p.A214T	Chr10: 103,990,540	Hom	Syndromic autosomal recessive Cataract-11	AR/AD	CD19	50 %	6–32 %	Citrulline	5.4	9–42
					Multiple types of Cataract-11		CD4	39 %	35–64 %	isoleucine	10.5	24–105
					Multiple subtypes of anterior segment dysgenesis-1		CD8	9 %	12–28 %	Leucine	24.4	48–205
PRF1 ENST00000441259.1 NM_005041	Exon 3	c.853_855delAAG p.K285del	Chr10: 72,358,622-72,358,624	Het	Familial hemophagocytic lymphohistiocytosis-2	AR				threonine	30.3	threonine
					Aplastic anemia	–				Tyrosine	14.9	Tyrosine
					Non-Hodgkin Lymphoma	–				valine	44.6	valine
USH2A ENST00000307340.3 NM_206933	Exon 54	c.10613G > A p.R3538Q	Chr1: 215,955,511	Het	Retinitis pigmentosa-39	AR				**3 Months**		
					Usher syndrome-type 2 A	AR				Alanine	176	195–560
										valine	108	123–310
										Glutamine	734	345–685
										lysine	62	80–240
										Phenylalanine	29	32–85
										glycine	103	105–413

Human whole exome enrichment was performed by NGS using Twist Human Core Exome Plus Kit, and the library was sequenced on an Illumina platform with a raw coverage of 266×, performed by CeGaT GmbH, Germany. Nearly all exons and flanking 10 bp were detected and analyzed. Detected variations include single-point mutations and small indels (within 20 bp).

**- Metabolic Tests** show Elevated alpha-fetoprotein (239, normal <8.5) at 2 months and Elevated PTH (251.2, normal 9–94) at 5 months.

This test of Amino acid analysis at 2 and 3 months showed valine deficiency (Table 1).

The acylcarnitine profile in plasma test was performed at 2 months, and all the analytes were in the normal range.

## 3 Discussion

The patient presents with classical features of Vici syndrome, including developmental delay, albinism, cataracts, agenesis of the corpus callosum, hypotonia, and immune system anomalies. Multiple stones in the kidney, bladder, and gallbladder are notable.

Vici syndrome presents with comprehensive manifestations indicative of a hereditary multisystem disorder reported in humans. It consistently manifests in the first months of life. In addition to the five primary features of the syndrome, such as agenesis of the corpus callosum, cataracts, cardiomyopathy, hypopigmentation, and combined immunodeficiency, other varied manifestations of the disease have also been reported, suggesting that almost every organ system can be involved [4]. The patient's complex clinical picture highlights the need for a multidisciplinary approach to managing Vici syndrome.

Around 90 % of patients with Vici syndrome exhibit cardiac involvement, with cardiomyopathy, one of the five key diagnostic features, identified in approximately 80 % of cases. Minor congenital heart defects, including persistent foramen oval and atrial septal defects, are found in roughly 10 % of patients. While cardiomyopathy usually manifests early in life, it can also present later in childhood, and intermittent cardiac function deterioration during illnesses has been observed The cardiac findings, including a pedunculated mass resembling a rhabdomyoma, add a special factor to this case. The mass, which subsequently regressed spontaneously, coupled with the patient's stable cardiac function and laboratory findings, argues against a thrombus. Given these features and the initial echocardiographic findings, the mass resembled a cardiac rhabdomyoma, a common occurrence in children of this age group. Notably, the pedunculated morphology of Rhabdomyoma is exceedingly rare in children and has not been previously documented in association with Vici syndrome.

This is notable because these tumors are also a characteristic feature of tuberous sclerosis complex (TSC), a disorder associated with mutations in the TSC1 gene. The similarities in clinical phenotypes between EPG5-related disorders and TSC1-related disorders may be due to the involvement of TSC1 in upstream autophagy regulation. Autosomal recessive mutations in the EPG5 gene, impair the late stages of autophagy by disrupting autophagosome-lysosome fusion. Loss-of-function mutations in TSC1 or TSC2 genes lead to constitutive mTORC1 activation, blocking autophagic flux at multiple stages. Disrupted interactions between the lysosomal pathway and autophagosome biogenesis may further compromise the coordinated regulation of these cellular compartments. While the specific molecular pathways affected differ, both conditions ultimately lead to impaired cellular waste removal and potentially abnormal cell growth, contributing to the formation of these tumors.

Sensorineural hearing loss has been reported in multiple cases of this syndrome, and this finding was also observed in our study [[5], [6], [7]]. Compared to previous recordings, the absence of bilateral ABR suggests a potential neurodegenerative disorder affecting the retro-cochlear pathway in this patient.

According to the available literature, 79 published case reports have been made since Vici syndrome was first reported in 1988. Based on the available articles, the case we report is the second case of Vici syndrome reported in Iran. The table below summarizes the clinical symptoms of these cases and the frequency of these symptoms among the 79 known cases as percentages [2]. Additionally, a column indicates whether our patient's symptoms align with those of other reported cases, marked as positive or negative. As can be seen, our case matches other known cases in all symptoms except for the absence of a cleft palate, which is one of the features of this syndrome (Table 2).

**Table 2:** The difference between our case report and other reported cases.

Feature	Our patient	Positive	Negative	Not reported	n (%)
Recurrent infections	+	77	−	1	77/78 (98.7)
Corpus callosum agenesis	+	76	−	2	76/78 (97.4)
Profound developmental delay	+	76	1	1	76/78 (97.4)
Cutaneous manifestations	+	75	3	−	75/78 (96.2)
Immune system involvement	+	60	16	2	60/78 (76.9)
Cardiomyopathy	−	51	18	9	51/78 (65.4)
Cataract	+	49	25	4	49/78 (62.8)
Microcephaly	+	45	15	18	45/78 (57.7)
Hypotonia	+	37	−	41	37/78 (47.4)
Seizures	+	26	16	36	26/78 (33.3)
Growth retardation	+	24	−	54	24/78 (30.8)

The symptoms of this syndrome manifest as unilateral or bilateral cataracts, optic neuropathy, nystagmus, and ptosis. Brain MRI is a tool used to diagnose the corpus callosum agenesis. However, no further MRI imaging has been performed due to multiple diagnostic evaluations by various specialists and genetic testing serving as the confirmatory factor for this genetic disorder in this case [2].

The detected homozygous nonsense variant in the EPG5 gene has not been previously reported for its pathogenicity. However, a known disease mechanism is a null variant in the EPG5 gene (including the nonsense variant). Multiple lines of silico computational analysis (MutationTester and CADD) support the variant's deleterious effect on the gene or gene product(s). Based on ACMG guidelines, this variant can be classified as a likely pathogenic variant.

The detected homozygous missense variant in the PITX3 gene has not previously been reported for its pathogenicity. The prediction of computational tools (MutationTester, SIFT, and PolyPhen) suggests that the variant has no impact on the gene or gene product(s). Based on ACMG guidelines, this variant can be classified as a Variant of Uncertain Significance. Two variants were detected in the PRF1 and USH2A genes in this patient. These genes are not related to the patient's phenotype.

In immunological Tests, the patient has a reduced percentage of overall T cells (CD3) and cytotoxic T cells (CD8), which might affect their immune response capability. An elevated percentage of B cells (CD19) could indicate an abnormal immune response or other underlying conditions (Table 1). These findings could point to various immunological disorders or conditions. Combined immunodeficiency, a key diagnostic feature of Vici syndrome, shows considerable variability based on age, ranging from nearly normal to severely compromised immunity. Additionally, further immunological studies of this disease have been recommended [6].

Among the primary causes of death in early infancy for Vici syndrome cases are heart failure and sepsis. Studies show that individuals with Vici syndrome have a median survival time of 24 months. Therapeutic interventions for this group of patients are primarily supportive. Consanguinity is also a significant factor contributing to the development of this genetic disorder, and in our case, this familial relationship exists between the parents. In these patients, multi-organ dysfunction often leads to mortality. Recurrent infections, heart failure, and immunological issues cause further complications, necessitating continuous follow-up to ensure timely intervention and treatment when needed [8].

Mutations in EPG5 were identified as being associated with Vici syndrome. The Ectopic P granules protein five homologs (EPG5 (plays a crucial role in the autophagy pathway, encoding a key regulator of this process [9].

## 4 Conclusion

This case underscores the importance of early recognition and comprehensive management of Vici syndrome. Further studies are warranted to explore the genetic and phenotypic variability in patients with this rare disorder. Although there is no cure for Vici syndrome, management of the disease is supportive and aimed at alleviating symptoms. As some deficiencies may progress over time, it is necessary to perform certain tests, including EEG, ophthalmological evaluation with slit lamp examination, chest X-ray (CXR), cardiac assessment including echocardiography, and evaluations of immune function, thyroid, liver, and kidney. For immunodeficiency, intravenous immunoglobulin injections and regular antimicrobial prophylaxis may be required if necessary. Patients may also present with seizures, which might necessitate intervention with antiepileptic medications. If indicated, cataract surgery can be performed based on the severity of the condition. Additionally, if anemia develops, blood transfusion should be considered [3].

## Ethical statement

We have fully respected the rights of participants and stakeholders, adhering to all ethical principles related to medical research.

All data collection and information gathering processes were conducted with the informed consent of participants, ensuring their privacy.

## Funding and conflict of interest statement

The authors state that no financial or personal affiliation would affect the project in writing this article.

## CRediT authorship contribution statement

**Parsa Forouhar:** Writing – review & editing, Writing – original draft, Supervision, Project administration, Investigation, Data curation. **Mohammad Amin Eghtedari:** Writing – review & editing. **Maryam Taraz:** Writing – review & editing, Investigation. **Mahsa Abdullahpour:** Writing – review & editing, Investigation. **Mohammad Mehrpouyan:** Writing – review & editing, Investigation. **Zhale Askarzade:** Writing – review & editing.

## Declaration of competing interest

The authors state that no financial or personal affiliation would affect the project in writing this article.

## Data Availability

The data that has been used is confidential.
